# Contralateral parenchymal enhancement on breast MRI before and during neoadjuvant endocrine therapy in relation to the preoperative endocrine prognostic index

**DOI:** 10.1007/s00330-020-07058-3

**Published:** 2020-07-20

**Authors:** Max A. A. Ragusi, Claudette E. Loo, Bas H. M. van der Velden, Jelle Wesseling, Sabine C. Linn, Regina G. Beets-Tan, Sjoerd G. Elias, Kenneth G. A. Gilhuijs

**Affiliations:** 1 Department of Radiology/Image Sciences Institute, University Medical Center Utrecht, Utrecht University, Heidelberglaan 100, 3584 CX Utrecht, The Netherlands; 2 Department of Radiology, The Netherlands Cancer Institute – Antoni van Leeuwenhoek Hospital, Plesmanlaan 121, 1066 CX Amsterdam, The Netherlands; 3 Department of Pathology, The Netherlands Cancer Institute – Antoni van Leeuwenhoek Hospital, Plesmanlaan 121, 1066 CX Amsterdam, The Netherlands; 4 Department of Medical Oncology, The Netherlands Cancer Institute – Antoni van Leeuwenhoek Hospital, Plesmanlaan 121, 1066 CX Amsterdam, The Netherlands; 5 Department of Epidemiology, Julius Center for Health Sciences and Primary Care, University Medical Center Utrecht, Utrecht University, Universiteitsweg 100, 3584 CG Utrecht, The Netherlands

**Keywords:** Breast neoplasms, Neoadjuvant therapy, Parenchymal tissue, Magnetic resonance imaging

## Abstract

**Objectives:**

To investigate whether contralateral parenchymal enhancement (CPE) on MRI during neoadjuvant endocrine therapy (NET) is associated with the preoperative endocrine prognostic index (PEPI) of ER+/HER2− breast cancer.

**Methods:**

This retrospective observational cohort study included 40 unilateral ER+/HER2− breast cancer patients treated with NET. Patients received NET for 6 to 9 months with MRI response monitoring after 3 and/or 6 months. PEPI was used as endpoint. PEPI is based on surgery-derived pathology (pT- and pN-stage, Ki67, and ER-status) and stratifies patients in three groups with distinct prognoses. Mixed effects and ROC analysis were performed to investigate whether CPE was associated with PEPI and to assess discriminatory ability.

**Results:**

The median patient age was 61 (interquartile interval: 52, 69). Twelve patients had PEPI-1 (good prognosis), 15 PEPI-2 (intermediate), and 13 PEPI-3 (poor). High pretreatment CPE was associated with PEPI-3: pretreatment CPE was 39.4% higher on average (95% CI = 1.3, 91.9%; *p* = .047) compared with PEPI-1. CPE decreased after 3 months in PEPI-2 and PEPI-3. The average reduction was 24.4% (95% CI = 2.6, 41.3%; *p* = .032) in PEPI-2 and 29.2% (95% CI = 7.8, 45.6%; *p* = .011) in PEPI-3 compared with baseline. Change in CPE was predictive of PEPI-1 vs PEPI-2+3 (AUC = 0.77; 95% CI = 0.57, 0.96).

**Conclusions:**

CPE during NET is associated with PEPI-group in ER+/HER2− breast cancer: a high pretreatment CPE and a decrease in CPE during NET were associated with a poor prognosis after NET on the basis of PEPI.

**Key Points:**

*• Change in contralateral breast parenchymal enhancement on MRI during neoadjuvant endocrine therapy distinguished between patients with a good and intermediate/poor prognosis at final pathology.*

*• Patients with a poor prognosis at final pathology showed higher baseline parenchymal enhancement on average compared to patients with a good prognosis.*

*• Patients with an intermediate/poor prognosis at final pathology showed a higher average reduction in parenchymal enhancement after 3 months of neoadjuvant endocrine therapy.*

**Electronic supplementary material:**

The online version of this article (10.1007/s00330-020-07058-3) contains supplementary material, which is available to authorized users.

## Introduction

A positive estrogen receptor (ER) in breast cancer determines if patients should receive endocrine treatment. However, not all patients with ER+ breast cancer benefit from endocrine treatment: 40–50% relapse after adjuvant endocrine therapy [1] and 50–70% show a clinical response after neoadjuvant endocrine therapy (NET) [1–3]. A more accurate prediction whether endocrine treatment will be effective would benefit these patients, and allow for better selection and personalization of endocrine treatment.

Early prediction of NET efficacy could be used to personalize the course of treatment, i.e., expedite surgery or switch to neoadjuvant chemotherapy (NAC) in poor responders.

Typically, response monitoring during neoadjuvant therapy is performed with imaging. Magnetic resonance imaging (MRI) of the breast is the most accurate and recommended modality [4, 5]. Several MRI features have been identified as predictors of tumor response during NAC [6–11]. However, research regarding response monitoring in NET is limited [12, 13].

A potential predictor of endocrine treatment efficacy is contralateral parenchymal enhancement (CPE). CPE is a quantitative measure of the relative late parenchymal enhancement of the healthy breast on MRI [14, 15] and differs from background parenchymal enhancement (BPE), which is a qualitative measure of early parenchymal enhancement. CPE is calculated as the mean of the top-10% relatively most enhancing voxels. A high CPE was shown to be associated with improved survival in unilateral ER+ human epidermal growth factor 2 receptor–negative (HER2−) breast cancer patients after adjuvant endocrine therapy [14, 15]. If CPE is also associated with NET efficacy, it could be used to personalize the course of NET in breast cancer patients.

It is hypothesized that the contralateral breast represents the diseased breast before tumorigenesis [14], or may represent systemic (inflammatory) effects induced by the tumor [16]. CPE represents the highest delayed enhancement in healthy fibroglandular tissue. CPE might be affected by hormonal activity, as parenchymal enhancement varies during the menstrual cycle [17]. The underlying biological reason for the observed association between CPE and survival after endocrine treatment is unknown, but was demonstrated in two independent studies [14, 15]. Investigating the behavior of CPE during NET might not only provide a tool for the personalization of NET but could also provide insights into the underlying biological mechanisms.

Pathologic complete response (pCR) after neoadjuvant treatment is a controversial surrogate endpoint of prognosis in ER+/HER2− breast cancer [18, 19]. pCR is poorly associated with prognosis in ER+/HER2−, and rate of pCR is low in both NAC and NET (about 7.5% and < 10% respectively) [18–20]. To understand how tumor response after NET is related to prognosis, the preoperative endocrine prognostic index (PEPI) was developed [21]. PEPI is derived from the surgical excision specimen after NET and is based on pT- and pN-stage, Ki67 index, and ER-status. PEPI stratifies patients in three groups with distinct prognoses: PEPI-1 has the most favorable prognosis, whereas PEPI-3 has the poorest prognosis. PEPI can be used to personalize treatment after NET: patients with PEPI-1 have such a favorable prognosis that adjuvant endocrine monotherapy could suffice, whereas appropriate adjuvant treatment should be considered for PEPI-2 and PEPI-3 patients [21, 22]. PEPI was validated in the IMPACT trial [21] and the ACOSOG Z1031 trial [22].

In this study, we present a retrospective observational cohort study of patients with invasive unilateral ER+/HER2− breast cancer treated with NET. The aim was to determine whether pretreatment CPE or changes in CPE during treatment are associated with prognosis (on the basis of PEPI) after NET.

## Materials and methods

### Patient cohort and treatment

This retrospective explorative observational cohort study was approved by the Institutional Review Board of the Antoni van Leeuwenhoek Hospital and the requirement for informed consent was waived. All female patients with pathologically proven unilateral ER+/HER2− breast cancer diagnosed between January 2013 and December 2017 and eligible for NET according to the hospital’s institutional guidelines were included (*n* = 44 ). Additionally, the contralateral healthy breast did not contain any additional lesions (benign or malignant); a healthy breast is required for the calculation of CPE. The guidelines for NET are as follows: if breast-conserving surgery (BCS) cannot be performed or to reduce risk of irradicality at surgery (e.g., in the case of an invasive lobular carcinoma) for strongly ER+ (≥ 50%)/HER2− tumors, NET is recommended for a duration of 6 to 9 months. Additionally, there should be no indication for NAC: the tumor is ≤ 30 mm and there is ≤ 1 suspicious lymph node in combination with a low-risk Mammaprint 70-gene signature, or if there is excess comorbidity. This is decided during a multidisciplinary meeting. NET consisted of tamoxifen in premenopausal patients and aromatase inhibitors (AI) in postmenopausal patients. Clinical response is assessed after 3 and 6 months with ultrasound or MRI. If the tumor is stable or progressive, surgery is performed or the endocrine treatment is switched; otherwise, the duration of NET is completed.

**Fig. 1:**
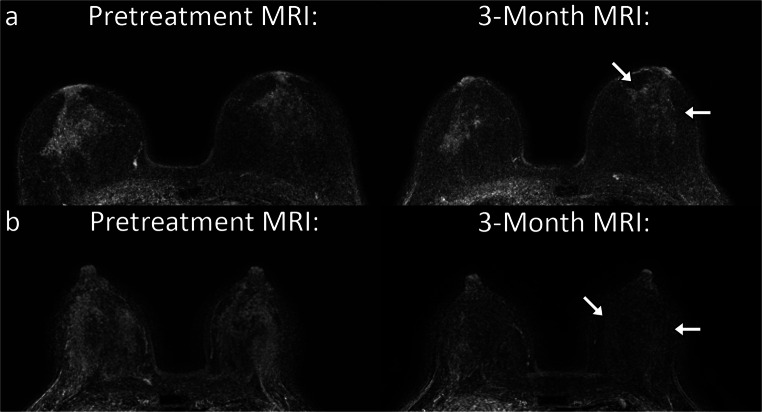
Pretreatment and 3-month follow-up maximum intensity projection images (slab = 25) of the subtraction of the late and early post-contrast series. The top row (**a**) shows the images of a 65-year-old patient with a T2N0M0 lobular carcinoma in the right breast. Note the persistence of parenchymal enhancement after 3 months of neoadjuvant endocrine therapy on the subtraction images of the late and early post-contrast series (arrows). The tumor was PEPI-1 (good prognosis) at surgical pathology. The bottom row (**b**) shows the images of a 45-year-old patient with a T1cN1M0 ductal carcinoma in the right breast. Note the decrease in parenchymal enhancement after 3 months of neoadjuvant endocrine therapy on the subtraction images of the late and early post-contrast series (arrows). The tumor ended up being PEPI-3 (poor prognosis) at surgical pathology

### MR imaging

MR images were acquired on a 1.5-T or 3-T imaging unit (Achieva, Philips) using a dedicated 4-, 7-, or 16-element SENSE breast coil (Philips). First, an unenhanced T1-weighted sequence with fat suppression was performed. Following intravenous injection of gadolinium-containing contrast (0.1 mmol/kg, Dotarem, Guerbet), dynamic contrast series were obtained with early timing 90 s post-contrast injection and late timing 360 s post-contrast injection. One of two sets of imaging parameters were used: acquisition time 60 s or 70 s, ratio of repetition time/echo time 3.7/1.9 or 4.3/1.8, flip angle 10°, voxel sizes 0.618 × 0.618 × 1.150 mm^3^ or 0.885 × 0.885 × 0.900 mm^3^, and a field of view 400 mm. For nine patients, the pretreatment MRI was performed in a referring hospital. Details of the imaging parameters are provided in the Supplement Materials 1.

### Contralateral parenchymal enhancement

MRIs were processed using a previously reported method [14, 15]. Image processing was implemented using Python version 3.7 (Python Software Foundation) with the SimpleITK (version 1.2.0) library [23]. In short, field inhomogeneity was corrected. The breast area was segmented on pre-contrast non-fat-suppressed T1-weighted images and parenchymal tissue was segmented using fuzzy-C means clustering. Early and late post-contrast series were registered to the pre-contrast series to compensate for patient motion. Images with uncorrectable motion artifacts were excluded (*n* = 2). Relative parenchymal enhancement was calculated at each voxel within the healthy parenchymal tissue by subtracting the early parenchymal enhancement from the late parenchymal enhancement, and dividing this by the early parenchymal enhancement: (*S*_late_ − S_early_) / *S*_early_, where *S* represents the signal intensity at the corresponding time point. CPE is calculated as the mean of the top-10% most relatively enhancing voxels and is a measure of the relative late parenchymal enhancement. CPE is a dimensionless number and can be compared within and between patients.

### Endpoint

PEPI was used as a surrogate endpoint of prognosis [21, 22]. PEPI is derived from the surgical excision specimen and is based on the following characteristics: pT- and pN-stage, Ki67 proliferation index, and ER-status [21]. Risk points are assigned based on these four characteristics. The total risk score (on a scale of 0–12) stratifies the patient in one of three prognostic groups: groups 1 (0 points), 2 (1–3 points), and 3 (≥ 4 points). Patients with unavailable PEPI score due to insufficient tumor material in the surgical excision specimen were excluded (*n* = 2). Additionally, the pCR results are provided. pCR was defined as the absence of invasive disease (ypT0/is N0) [24]. Pathologic partial or non-response was based on reduction of tumor cellularity using the Pinder classification [25].

### Statistical methods

Standard descriptive statistics were used to describe the study population. Pretreatment CPE tertile values were used to split patients in three patient groups for baseline characteristics (baseline characteristics split according to PEPI-group is provided in the Supplement Materials 2). Descriptive statistics are reported as median (interquartile interval [IQI]). A multivariable linear mixed model (LMM) was fit to investigate whether pretreatment CPE or changes in CPE over time are associated with PEPI-group. An LMM is a statistically efficient method to analyze repeated measurements within a patient [26]. In the multivariable analysis, CPE was modeled as a function of time (both categorically at 0, 3, and 6 months and continuously), PEPI-group, and the interaction between PEPI-group and time. An interaction between PEPI-group and time allows a possible change of CPE over time to differ between PEPI-groups. CPE was adjusted for baseline differences in age and type of NET regimen. The differences in pretreatment CPE and changes in CPE during NET between the PEPI-groups can be derived from the same model. To account for repeated measurements, we included random intercepts for patients. CPE was log-transformed to improve model fit. Nested models were compared using maximum likelihood estimation. Effect estimates were based on restricted maximum likelihood with Satterthwaite’s approximations to the degrees of freedom.

Univariable and multivariable logistic regressions were performed to set up models to assess the discriminatory ability of pretreatment CPE and change in CPE (slope). To assess discriminatory ability between PEPI-1 and PEPI-2+3, and between PEPI-1+2 and PEPI-3, the area under the curve (AUC) was calculated using the receiver operating characteristic (ROC) analysis. The ROC analyses were assessed by comparing the underlying logistic regression models using the likelihood ratio test.

Statistical analyses were performed using R version 3.4.4 (R Foundation for Statistical Computing) and the LMM was fit using the “lme4” (version 1.1.21) [27] and “lmerTest” (version 3.1.0) [28] packages available in R. Coefficient estimates are reported with their corresponding 95% confidence intervals (CI). A two-tailed *p* < .05 was considered to represent statistical significance. The study is reported following the STROBE guidelines [29].

## Results

### Patient cohort

Patient, tumor, and treatment characteristics are summarized in Table 1. Forty patients were included and 81 CPE measurements were available for analysis. The median patient age was 61 years (IQI = 52, 69). Characteristics between these baseline groups were balanced for age, tumor histology, cN-stage, ER-percentage, and pretreatment Ki67 index (Table 1). Some unbalance was noted in the cT-stage and tumor grade: the group with high baseline CPE (third tertile) showed relatively more prognostic favorable characteristics compared to the groups with lower baseline CPE (e.g., more T1c and grade 1). Premenopausal patients seem overrepresented in the second tertile group, which is reflected in the distribution of NET regimen: more patients in this group received tamoxifen. There was a difference in CPE of + 28.5% (95% CI = − 48.6, 65.6%, *p* = .358) in premenopausal patients compared with postmenopausal patients.

**Table 1:** Patient, tumor, and treatment characteristics of the entire cohort and according to pretreatment CPE tertile values

Characteristics	Overall (*n* = 40)	Baseline CPE, tertile 1 (*n* = 13)	Baseline CPE, tertile 2 (*n* = 12)	Baseline CPE, tertile 3 (*n* = 13)
CPE				
Median (range)	0.29 (0.16–0.80)	0.21 (0.16–0.26)	0.30 (0.27–0.37)	0.48 (0.39–0.80)
Age (years)				
Median (IQI)	61 (52–69)	61 (54–70)	63 (48–69)	62 (52–69)
Menopausal status				
Premenopausal	10 (25.0%)	2 (15.4%)	5 (41.7%)	3 (23.1%)
Tumor size on pretreatment MRI (mm)				
Median (IQI)	28 (26–41)	30 (25–41)	28 (27–29)	36 (27–49)
cT-stage				
1c	8 (20.0%)	1 (7.7%)	2 (16.7%)	5 (38.5%)
2	24 (60.0%)	9 (69.2%)	8 (66.7%)	5 (38.5%)
3	6 (15.0%)	2 (15.4%)	1 (8.3%)	3 (23.1%)
4b	2 (5.0%)	1 (0%)	1 (8.3%)	0 (0%)
cN-stage				
Negative	30 (75.0%)	11 (84.6%)	7 (58.3%)	10 (76.9%)
Positive	10 (25.0%)	2 (15.4%)	5 (41.7%)	3 (23.1%)
Tumor grade				
1	6 (15.4%)	0 (0%)	2 (16.7%)	4 (33.3%)
2	27 (69.2%)	9 (69.2%)	9 (75.0%)	7 (58.3%)
3	6 (15.4%)	4 (30.8%)	1 (8.3%)	1 (8.3%)
Unknown	1	0	0	1
Tumor histology				
IDC	24 (60.0%)	7 (53.8%)	8 (66.7%)	8 (61.5%)
ILC	12 (30.0%)	4 (30.8%)	2 (16.7%)	5 (38.5%)
Other	4 (10.0%)	2 (15.4%)	2 (16.7%)	0 (0%)
ER-percentage				
Median (IQI)	100 (95–100)	100 (90–100)	100 (100–100)	100 (95–100)
Ki67				
Pretreatment (IQI)	10 (5, 16.3)	10 (5, 20)	11.3 (10, 16.3)	7.5 (2, 10)
Post-treatment (IQI)	5 (1, 5)	2 (1, 10)	3 (1, 5)	5 (1, 5)
NET duration (months)				
Median (IQI)	7.2 (6.6–8.0)	7.0 (6.6–7.6)	7.5 (6.7–8.7)	7.2 (6.6–8.7)
Type of NET				
Combination	5 (12.5%)	0 (0%)	2 (16.7%)	3 (23.1%)
Aromatase inhibitor	23 (57.5%)	10 (76.9%)	5 (41.7%)	7 (53.8%)
Tamoxifen	12 (30.0%)	3 (23.1%)	5 (41.7%)	3 (23.1%)

Unless otherwise specified, data are number of patients, with percentages in parentheses. The discrepancy in overall and grouped total patient numbers is due to unavailability of baseline CPE for two patients. *CPE* contralateral parenchymal enhancement, *IQI* interquartile interval, *IDC* invasive ductal carcinoma, *ILC* invasive lobular carcinoma, *ER* estrogen receptor, *NET* neoadjuvant endocrine therapy

Six patients (15%, 6/40) had progressive disease at 3 months of follow-up: one patient switched treatment regimen (tamoxifen to AI), and in five patients, surgery was expedited. The remaining 34 patients were considered (partial) responders at 3-month follow-up and completed the full duration of NET. The median duration of NET was 7.2 months (IQI = 6.6 to 8.0). After NET, 12 patients had a good prognosis (PEPI-1), 15 patients had an intermediate prognosis (PEPI-2), and 13 patients had a poor prognosis (PEPI-3). For the six patients who were clinically considered to be non-responders after 3 months, the distribution of PEPI scores was one patient with PEPI-1 (the patient who switched regimen), two patients with PEPI-2, and three patients with PEPI-3. One patient (2.5%) showed a pCR at surgical pathology, and five patients (12.5%) showed no pathologic response. The remaining 34 patients (85%) showed a partial pathologic response after NET (Supplemental Materials 2). The five patients who showed no pathologic response related to the PEPI-2 or PEPI-3 group.

### Pretreatment CPE and changes in CPE are associated with the PEPI-group

#### Pretreatment CPE and PEPI-group

In the multivariable analysis, pretreatment CPE was on average higher in the group with a poor prognosis after NET (PEPI-3), independent of age and type of NET by 39.4% (95% CI = 1.3, 91.9%; *p* = .047, Table 2). An average difference of + 11.4% (95% CI = − 17.5, 50.4%; *p* = .474) was observed in PEPI-2 (intermediate prognosis).

**Table 2:** Multivariable estimates of differences in CPE according to PEPI-group in time

Variables	%-change in CPE	*p* value
Baseline CPE		
PEPI-1	Ref	
PEPI-2	11.4 (− 17.5, 50.4)	.474
PEPI-3	39.4 (1.3, 91.9)	.047
Change in CPE for PEPI-1 over time		
Baseline	Ref	
After 3 months of NET	27.6 (− 0.1, 62.9)	.051
After 6 months of NET	29.4 (0.0, 67.4)	.050
Per month*	4.6 (0.3, 9.0)	.042
Change in CPE for PEPI-2 over time		
Baseline	Ref	
After 3 months of NET	− 24.4 (− 41.3, − 2.6)	.032
After 6 months of NET	− 12.8 (− 30.7, 9.6)	.232
Per month*	− 2.7 (− 6.4, 1.4)	.172
Change in CPE for PEPI-3 over time		
Baseline	Ref	
After 3 months of NET	− 29.2 (− 45.6, − 7.8)	.011
After 6 months of NET	− 23.7 (− 46.6, 9.1)	.135
Per month*	− 6.0 (− 11.6, 0.1)	.052

Data in parentheses are 95% confidence intervals. Estimates for %-change in CPE with the corresponding PEPI-group as reference group (e.g., change after 3 months in PEPI-3 is − 29.2% relative to baseline CPE of PEPI-3). The interaction term (i.e., change in CPE over time dependent on PEPI-group) significantly improved the model (*p* = .004). Results from the model with time as a linear variable are marked with a “*”. Estimates were adjusted for age and type of NET. *Ref* reference group, *CPE* contralateral parenchymal enhancement, *PEPI* preoperative endocrine prognostic index, *NET* neoadjuvant endocrine therapy

#### Change in CPE over time and PEPI-group

**Fig. 2:**
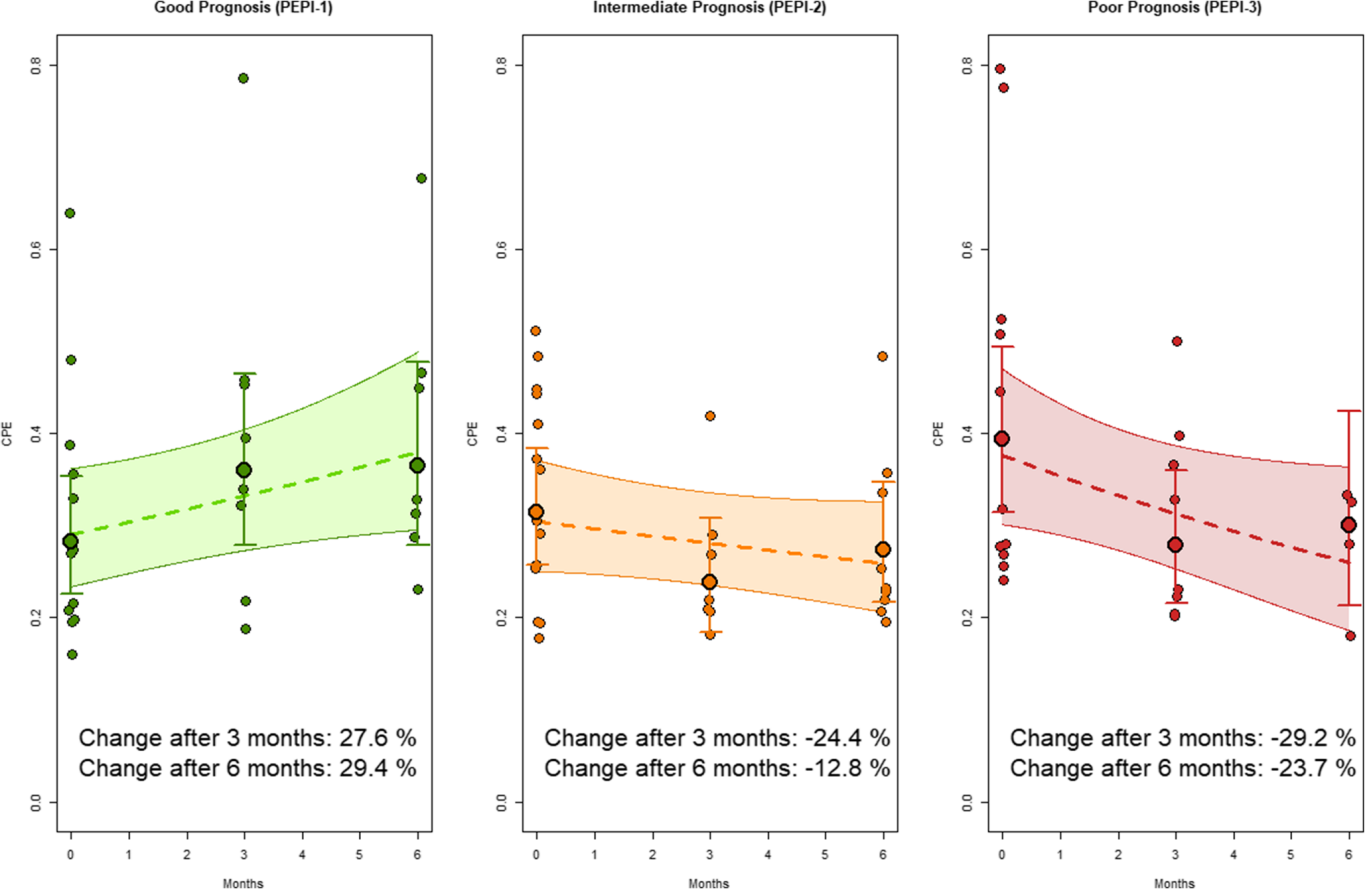
Overview of the change in CPE over time for the different PEPI-groups during neoadjuvant endocrine therapy at different time points: pretreatment (0 months), after 3 months, and after 6 months and per month. Individual CPE values are shown as dots. Modeled CPE is shown over time, with time modeled categorically (points with the 95% CI as whiskers) and with time modeled linearly (dashed line with and the shaded areas as the 95% CI). CPE increased over time in patients with a good prognosis after NET (PEPI-1), whereas it decreased over time in patients with an intermediate or poor prognosis after NET (PEPI-2 and PEPI-3)

Change in CPE over time during NET was significantly different between the PEPI-groups (*p*_interaction_ = .004). In the multivariable analysis, CPE increased over time in patients with a good prognosis (PEPI-1) and decreased in patients with a poor prognosis (PEPI-2 and PEPI-3), independent of age and type of NET. In the model with time modeled categorically, most change in CPE occurred during the first 3 months of NET: CPE increased by 27.6% on average (95% CI = − 0.1, 62.9%; *p* = .051) in PEPI-1 compared with baseline, decreased by 24.4% (95% CI = 2.8, 41.3%; *p* = .032) in PEPI-2, and decreased by 29.2% (95% CI = 7.8, 45.6%; *p* = .011) in PEPI-3 (Table 2). A representative example is shown in Fig. 1. CPE increased by 29.4% on average (95% CI = 0.0, 67.4%; *p* = .050) relative to baseline in PEPI-1 after 6 months. An average difference of − 12.8% (95% CI = − 9.6, 30.7; *p* = .232) was observed in PEPI-2 and − 23.7% (95% CI = − 9.1, 46.6%; *p* = .135) in PEPI-3 (Fig. 2). In the multivariable analysis with time modeled linearly, CPE increased on average in PEPI-1 by 4.6% (95% CI = 0.3, 9.0%; *p* = .042) each month, whereas an average difference of − 2.7% (95% CI = − 1.4, 6.4; *p* = .172) and − 6.0% (95% CI = 0.1, 11.6%; *p* = .052) was observed in PEPI-2 and PEPI-3, respectively, independent of age and type of NET.

### Ability of pre- and during-treatment CPE to discriminate between PEPI-groups

Twenty-nine patients were available for ROC analysis to discriminate between PEPI-groups using pretreatment CPE and change in CPE during treatment. Pretreatment CPE was not able to discriminate between the PEPI-groups: the AUC to distinguish between PEPI-1 and PEPI-2+3 was 0.65 (95% CI = 0.43, 0.87), and 0.67 (95% CI = 0.43, 0.90) to distinguish between PEPI-1+2 and PEPI-3. However, change in CPE was able to discriminate between the PEPI-groups: the AUC to distinguish between PEPI-1 and PEPI-2+3 was 0.77 (95% CI = 0.57, 0.96), and 0.77 (95% CI = 0.54, 0.99) for PEPI-1+2 vs PEPI-3. Differences in pretreatment CPE were not useful in discriminating between the different PEPI-groups as the AUCs based on both pretreatment CPE and change in CPE during treatment were comparable with the AUCs based solely on the change in CPE: the AUC based on pretreatment and change in CPE was 0.77 (95% CI = 0.59, 0.94; *p* = .307) for PEPI-1 vs PEPI-2+3 and 0.81 for PEPI-1+2 vs PEPI-3 (95% CI = 0.63, 0.96; *p* = .325).

## Discussion

In this retrospective single-center observational cohort study, we showed that pretreatment CPE, a quantitative measure of relative late parenchymal enhancement on MRI, and change in CPE during NET were associated with PEPI-group in the post-treatment surgical specimen: a high pretreatment CPE and a decrease in CPE during NET were associated with a higher PEPI-group (poor prognosis).

Research regarding response imaging during NET is limited. Our results are in agreement with the findings of Hilal et al, who found that high pretreatment BPE, classified according to the BI-RADS lexicon, was associated with non-responders after NET [13]. In the NAC setting, BPE has been linked to several treatment outcomes [6]: a high BPE before start of NAC was associated with worse recurrence-free survival (RFS) [30], while a decrease in BPE during NAC was associated with pCR [31–33].

While a decrease in parenchymal enhancement on MRI during NAC is reported to be associated with pCR, in our study, a decrease in CPE was associated with an unfavorable prognosis after NET. Perhaps one would expect parenchymal enhancement to decrease in patients with effective endocrine treatment due to depressed hormonal activity, as BPE is increased during physiological hormonal activity [34] or during hormone replacement therapy [35, 36]. BPE was associated with increased microvessel density [37]: persistent or increased parenchymal enhancement during NET might reflect increased perfusion and better drug delivery. CPE was not associated with percent staining of ER or progesterone receptor on immunohistochemistry, nor with genomic ER-pathway activity in the tumor [15, 38]. A different explanation for these opposing effects between the different neoadjuvant therapies might be due to different immunohistochemical subtypes of breast cancer. It is known that breast cancer is a heterogeneous disease with different prognoses, treatment, and imaging characteristics, especially in ER+/HER2− breast cancer [39]. Differences in tumor biology and treatment mechanisms (cytotoxic chemotherapy vs antiproliferative endocrine therapy) could have had different systemic effects on the fibroglandular tissue, which could lead to differences in the behavior of parenchymal enhancement. Without a clear understanding of the biological basis of parenchymal enhancement and treatment efficacy, and the (dis)similarity between BPE and CPE, it is difficult to provide an explanation for these opposing findings between NAC and NET.

Although the changes in parenchymal enhancement are counterintuitive in the context of chemotherapy, a high CPE was previously associated with a favorable prognosis after adjuvant endocrine therapy [14, 15]. In our study, an increase of CPE is associated with a favorable prognosis after NET. In that sense, a high CPE after NET was also associated with a favorable prognosis (PEPI-1).

Remarkably, high pretreatment CPE was related to a poor prognosis (PEPI-3) at final pathology, whereas high CPE was previously shown to be related with improved overall and invasive disease-free survival after adjuvant endocrine therapy [14, 15]. The exact reason for this finding is unknown, although the difference might simply be due to different endpoints. Additionally, pretreatment CPE alone was not useful in distinguishing between the different PEPI-groups at final pathology.

PEPI was used as a surrogate endpoint of prognosis because pCR and change in tumor size are poorly associated with prognosis in ER+/HER2− breast cancer [18, 19]. Specifically for ER+/HER2− breast cancer, change in tumor size during NAC is a poor predictor of response and a poorly reproducible surrogate endpoint of survival [40, 41]. Change in tumor size during NAC yielded a non-significant AUC for the prediction of pCR in one study [42] and was not associated with survival after NAC in another study [39]. Additionally, clinical response during NET was not associated with survival [21]. In our study, change in CPE during NET was associated with prognosis (on the basis of PEPI) and performed similarly to other mid-treatment predictors of tumor response in ER+/HER2− breast cancer after NAC: change in CPE discriminated PEPI with an AUC of 0.77, and change in apparent diffusion coefficient discriminated pCR with an AUC of 0.76 [11]. To our knowledge, CPE is the first quantitative imaging feature that was observed to be associated with prognosis at final pathology after NET.

Our results support the hypothesis that the healthy breast contains information about endocrine treatment success for patients with unilateral ER+/HER2− breast cancer. CPE was reported to stratify patients within high-risk groups based on genomic assays (70-gene signature and 21-gene recurrence score) [43]. These results suggest that CPE contains prognostic information independent of these genomic assays and could potentially be used to further personalize treatment.

The main limitation of this study is its relatively small size, which is reflected in the wide CIs of the estimates, and limits the power to detect small effects. To account for the small population size, we took full advantage of the statistical efficiency of a linear mixed model for the repeated measurements analysis, and the association between CPE and prognosis after NET was strong enough to reach the a priori defined significance threshold of < .05. The association between survival and CPE was previously shown to reproduce between different MRI vendors and small differences in imaging parameters [15]. For nine patients, the pretreatment MRI was performed in the referring hospital on a different MRI vendor which could have led to variability in the CPE measurements. However, the flip angle and repetition time, being the imaging parameters with the most influence on intensity [44], were similar over the entire cohort. Despite the differences in parameters, CPE was observed to be significantly associated with PEPI. Additionally, exclusion of the nine referred patients did not influence the results. Although there is currently no consensus on the optimal duration of NET, recent clinical studies treat patients for up to 24 weeks (about 6 months) [20], as there is evidence that maximum tumor response may be reached after 6 to 7 months of NET [45]. In this study, patients received NET for a median duration of 7.2 months. The findings should be validated in a larger cohort to assess the discriminatory ability of CPE during NET. Lastly, an important step for the implementation of quantitative measurements of parenchymal enhancement is the development of software for use in clinical practice.

In conclusion, pretreatment and changes in contralateral parenchymal enhancement during neoadjuvant endocrine treatment were associated with PEPI-group in unilateral ER+/HER2− breast cancer patients: a high pretreatment CPE and a decrease in CPE during NET were associated with a poor prognosis after NET on the basis of PEPI. Future research will focus on the potential of CPE to assess endocrine treatment effectiveness.

## Electronic supplementary material

ESM 1 (DOCX 20 kb)

## Abbreviations

CPE: Contralateral parenchymal enhancement
ER: Estrogen receptor
HER2: Human epidermal growth factor receptor 2
NAC: Neoadjuvant chemotherapy
NET: Neoadjuvant endocrine therapy
PEPI: Preoperative endocrine prognostic index

## References

[1] Selli C, Dixon JM, Sims AH (2016). Accurate prediction of response to endocrine therapy in breast cancer patients: current and future biomarkers. Breast Cancer Res.

[2] Miller WR, Larionov A, Renshaw L (2009). Gene expression profiles differentiating between breast cancers clinically responsive or resistant to letrozole. J Clin Oncol.

[3] Fontein DBY, Charehbili A, Nortier JWR (2014). Efficacy of six month neoadjuvant endocrine therapy in postmenopausal, hormone receptor-positive breast cancer patients - a phase II trial. Eur J Cancer.

[4] Fowler AM, Mankoff DA, Joe BN (2017). Imaging neoadjuvant therapy response in breast cancer. Radiology.

[5] Eisenhauer EA, Therasse P, Bogaerts J (2009). New response evaluation criteria in solid tumours: revised RECIST guideline (version 1.1). Eur J Cancer.

[6] Liao GJ, Henze Bancroft LC, Strigel RM (2020). Background parenchymal enhancement on breast MRI: a comprehensive review. J Magn Reson Imaging.

[7] Gampenrieder SP, Peer A, Weismann C (2019). Radiologic complete response (rCR) in contrast-enhanced magnetic resonance imaging (CE-MRI) after neoadjuvant chemotherapy for early breast cancer predicts recurrence-free survival but not pathologic complete response (pCR). Breast Cancer Res.

[8] Santamaría G, Bargalló X, Fernández PL, Farrús B, Caparrós X, Velasco M (2017) Neoadjuvant systemic therapy in breast cancer: association of contrast-enhanced MR imaging findings, diffusion-weighted imaging findings, and tumor subtype with tumor response. Radiology 283:663–672 10.1148/radiol.2016160176 27875106

[9] Goorts B, Dreuning KMA, Houwers JB (2018). MRI-based response patterns during neoadjuvant chemotherapy can predict pathological (complete) response in patients with breast cancer. Breast Cancer Res.

[10] Shin SU, Cho N, Lee HB (2018). Neoadjuvant chemotherapy and surgery for breast cancer: preoperative MRI features associated with local recurrence. Radiology.

[11] Partridge SC, Zhang Z, Newitt DC (2018). Diffusion-weighted MRI findings predict pathologic response in neoadjuvant treatment of breast cancer: the ACRIN 6698 multicenter trial. Radiology.

[12] Takeda K, Kanao S, Okada T (2012). MRI evaluation of residual tumor size after neoadjuvant endocrine therapy vs. neoadjuvant chemotherapy. Eur J Radiol.

[13] Hilal T, Covington M, Kosiorek HE et al (2018) Breast MRI phenotype and background parenchymal enhancement may predict tumor response to neoadjuvant endocrine therapy. Breast J 24:1–5 10.1111/tbj.13101 30066421

[14] van der Velden BH, Dmitriev I, Loo CE, Pijnappel RM, Gilhuijs KG (2015) Association between parenchymal enhancement of the contralateral breast in dynamic contrast-enhanced MR imaging and outcome of patients with unilateral invasive breast cancer. Radiology 276:675–685 10.1148/radiol.15142192 25811614

[15] van der Velden BHM, Sutton EJ, Carbonaro LA, Pijnappel RM, Morris EA, Gilhuijs KGA (2018) Contralateral parenchymal enhancement on dynamic contrast-enhanced MRI reproduces as a biomarker of survival in ER-positive/HER2-negative breast cancer patients. Eur Radiol 28:4705–4716 10.1007/s00330-018-5470-7 PMC6182741 29736850

[16] Pierce BL, Ballard-Barbash R, Bernstein L (2009). Elevated biomarkers of inflammation are associated with reduced survival among breast cancer patients. J Clin Oncol.

[17] Dowsett M, Nielsen TO, A’Hern R (2011). Assessment of Ki67 in breast cancer: recommendations from the international Ki67 in breast cancer working group. J Natl Cancer Inst.

[18] Von Minckwitz G, Untch M, Blohmer JU (2012). Definition and impact of pathologic complete response on prognosis after neoadjuvant chemotherapy in various intrinsic breast cancer subtypes. J Clin Oncol.

[19] Cortazar P, Zhang L, Untch M (2014). Pathological complete response and long-term clinical benefit in breast cancer: the CTNeoBC pooled analysis. Lancet.

[20] Spring LM, Gupta A, Reynolds KL (2016). Neoadjuvant endocrine therapy for estrogen receptor-positive breast cancer: a systematic review and meta-analysis. JAMA Oncol.

[21] Ellis MJ, Tao Y, Luo J (2008). Outcome prediction for estrogen receptor-positive breast cancer based on postneoadjuvant endocrine therapy tumor characteristics. J Natl Cancer Inst.

[22] Ellis MJ, Suman VJ, Hoog J (2017). Ki67 proliferation index as a tool for chemotherapy decisions during and after neoadjuvant aromatase inhibitor treatment of breast cancer: results from the American College of Surgeons Oncology Group Z1031 trial (alliance). J Clin Oncol.

[23] Yaniv Z, Lowekamp BC, Johnson HJ, Beare R (2018). SimpleITK image-analysis notebooks: a collaborative environment for education and reproducible research. J Digit Imaging.

[24] Green MC, Buzdar AU, Smith T (2005). Weekly paclitaxel improves pathologic complete remission in operable breast cancer when compared with paclitaxel once every 3 weeks. J Clin Oncol.

[25] Pinder SE, Provenzano E, Earl H, Ellis IO (2007). Laboratory handling and histology reporting of breast specimens from patients who have received neoadjuvant chemotherapy. Histopathology.

[26] Verbeke G, Molenberghs G (2004). A review on linear mixed models for longitudinal data, possibly subject to dropout. Stat Model.

[27] Bates D, Mächler M, Bolker BM, Walker SC (2015) Fitting linear mixed-effects models using lme4. J Stat Softw 67:1–51

[28] Kuznetsova A, Brockhoff PB, Christensen RHB (2017) lmerTest package: tests in linear mixed effects models. J Stat Softw 82:1–26

[29] Vandenbroucke JP, von Elm E, Altman DG (2014). Strengthening the Reporting of Observational Studies in Epidemiology (STROBE): explanation and elaboration. Int J Surg.

[30] Choi JS, Ko ES, Ko EY, Han B-K, Nam SJ (2016) Background parenchymal enhancement on preoperative magnetic resonance imaging: association with recurrence-free survival in breast cancer patients treated with neoadjuvant chemotherapy. Medicine (United States) 95:e3000 10.1097/MD.0000000000003000 PMC4782905 26945421

[31] Chen JH, Yu HJ, Hsu C, Mehta RS, Carpenter PM, Su YM (2015) Background parenchymal enhancement of the contralateral normal breast: association with tumor response in breast cancer patients receiving neoadjuvant chemotherapy. Transl Oncol 8:204–209 10.1016/j.tranon.2015.04.001 PMC4487259 26055178

[32] You C, Gu Y, Peng W (2018). Decreased background parenchymal enhancement of the contralateral breast after two cycles of neoadjuvant chemotherapy is associated with tumor response in HER2-positive breast cancer. Acta Radiol.

[33] Preibsch H, Wanner L, Bahrs SD (2016). Background parenchymal enhancement in breast MRI before and after neoadjuvant chemotherapy: correlation with tumour response. Eur Radiol.

[34] Müller-Schimpfle M, Ohmenhaüser K, Stoll P, Dietz K, Claussen CD (1997) Menstrual cycle and age: influence on parenchymal contrast medium enhancement in MR imaging of the breast. Radiology 203:145–149 10.1148/radiology.203.1.9122383 9122383

[35] Delille J-P, Slanetz PJ, Yeh ED, Kopans DB, Halpern EF, Garrido L (2007) Hormone replacement therapy in postmenopausal women: breast tissue perfusion determined with MR imaging—initial observations. Radiology 235:36–41 10.1148/radiol.2351040012 15798166

[36] Pfleiderer SOR, Sachse S, Sauner D (2004). Changes in magnetic resonance mammography due to hormone replacement therapy. Breast Cancer Res.

[37] Sung JS, Corben AD, Brooks JD (2018). Histopathologic characteristics of background parenchymal enhancement (BPE) on breast MRI. Breast Cancer Res Treat.

[38] van der Velden BHM, Bismeijer T, Canisius S (2019). Are contralateral parenchymal enhancement on dynamic contrast-enhanced MRI and genomic ER-pathway activity in ER-positive/HER2-negative breast cancer related?. Eur J Radiol.

[39] Loo CE, Straver ME, Rodenhuis S (2011). Magnetic resonance imaging response monitoring of breast cancer during neoadjuvant chemotherapy: relevance of breast cancer subtype. J Clin Oncol.

[40] Boughdad S, Champion L, Becette V et al (2020) Early metabolic response of breast cancer to neoadjuvant endocrine therapy: comparison to morphological and pathological response. Cancer Imaging 20:1–9 10.1186/s40644-020-0287-4 PMC6986018 31992361

[41] Dowsett M, Ebbs SR, Dixon JM (2005). Biomarker changes during neoadjuvant anastrozole, tamoxifen, or the combination: influence of hormonal status and HER-2 in breast cancer - a study from the IMPACT trialists. J Clin Oncol.

[42] Drisis S, Metens T, Ignatiadis M, Stathopoulos K, Chao SL, Lemort M (2016) Quantitative DCE-MRI for prediction of pathological complete response following neoadjuvant treatment for locally advanced breast cancer: the impact of breast cancer subtypes on the diagnostic accuracy. Eur Radiol 26:1474–1484 10.1007/s00330-015-3948-0 26310583

[43] Van Der Velden BHM, Elias SG, Bismeijer T (2017). Complementary value of contralateral parenchymal enhancement on DCE-MRI to prognostic models and molecular assays in high-risk ER+/HER2−breast cancer. Clin Cancer Res.

[44] Haacke EM, Filleti CL, Gattu R (2007). New algorithm for quantifying vascular changes in dynamic contrast-enhanced MRI independent of absolute T1 values. Magn Reson Med.

[45] Yeo B, Dowsett M (2015). Neoadjuvant endocrine therapy: patient selection, treatment duration and surrogate endpoints. Breast.

