# Supplementary material for: Contralateral parenchymal enhancement on breast MRI before and during neoadjuvant endocrine therapy in relation to the preoperative endocrine prognostic index
Source: Eur Radiol. 2020 Jul 20;30(12):6740–8. doi: 10.1007/s00330-020-07058-3 (PMC7599193; doi:10.1007/s00330-020-07058-3)
Supplement: Supplementary file 1 — (DOCX 20 kb) [file 330_2020_7058_MOESM1_ESM.docx]

| **Supplemental Materials 1:** Imaging Parameters of the Baseline MRIs of the Referred Patients | | | | | | | | | | |
| --- | --- | --- | --- | --- | --- | --- | --- | --- | --- | --- |
| No. of patients | Manufacturer | Model | Field Strength (T) | Echo time (msec) | Repetition time (msec) | Flip angle (degrees) | Voxelsize (mm^3^) | Field of view (mm^2^) | Timing early scan (s) | Timing late scan (s) |
| 5 | Siemens | Aera | 1.5 | 1.7 | 4.5 | 10 | 1.125x1.25x1.5 | 360 | 70-80 | 330-360 |
| 1 | Siemens | Espree | 1.5 | 1.3 | 4.3 | 10 | 0.759x0.759x0.9 | 340 | 95 | 350 |
| 1 | Siemens | Avanto | 1.5 | 1.3 | 4.3 | 10 | 0.8x0.8x0.9 | 360 | 80 | 360 |
| 1 | Siemens | Skyra | 3 | 2.1 | 5 | 20 | 0.804x0.804x1.0 | 360 | 90 | 390 |
| 1 | Siemens | Prisma Fit | 3 | 2.5 | 5.7 | 15 | 0.865x0.865x1.1 | 360 | 70 | 330 |

| **Supplemental Materials 2: Patient, Tumor and Treatment Characteristic of Entire Cohort and according to PEPI-group** | | | | |
| --- | --- | --- | --- | --- |
| **Characteristics** | Overall (n=40) | PEPI-1 (n=12) | PEPI-2 (n=15) | PEPI-3 (n=13) |
| **Age (years)** |  |  |  |  |
| *Median (IQI)* | 61 (52-69) | 65 (54 – 72) | 59 (52 – 67) | 59 (48 – 70) |
| **Menopausal status (%)** |  |  |  |  |
| *Premenopausal* | 11 (27.5 %) | 2 (16.7 %) | 4 (26.7 %) | 5 (38.5 %) |
| **cT-stage (%)** |  |  |  |  |
| *1c* | 8 (20 %) | 3 (25 %) | 1 (6.7 %) | 4 (30.8 %) |
| *2* | 24 (60 %) | 8 (66.7 %) | 10 (66.7 %) | 6 (46.2 %) |
| *3* | 6 (15 %) | 0 (0 %) | 3 (20 %) | 3 (23.1 %) |
| *4b* | 2 (5 %) | 1 (8.3 %) | 1 (6.7 %) | 0 (0 %) |
| **Tumor size on pretreatment MRI (mm)** |  |  |  |  |
| *Median (IQI)* | 28 (26 – 41) | 27 (25 – 39) | 35 (28 – 40) | 27 (21 – 54) |
| **cN-stage (%)** |  |  |  |  |
| *Negative* | 30 (75 %) | 11 (91.7 %) | 13 (86.7 %) | 6 (46.2 %) |
| *Positive* | 10 (25 %) | 1 (8.3%) | 2 (13.3 %) | 7 (53.8%) |
| **Tumor grade (%)** |  |  |  |  |
| *1* | 6 (16.7 %) | 3 (25 %) | 1 (6.7 %) | 2 (16.7 %) |
| *2* | 27 (69.2 %) | 6 (50 %) | 12 (80 %) | 9 (75 %) |
| *3* | 6 (16.7 %) | 3 (25 %) | 2 (13.3 %) | 1 (8.3 %) |
| *Unknown* | 1 | 0 | 0 | 1 |
| **Tumor histology (%)** |  |  |  |  |
| *IDC* | 24 (60 %) | 8 (66.7 %) | 8 (53.3 %) | 8 (61.5 %) |
| *ILC* | 12 (30 %) | 2 (16.7 %) | 5 (33.3 %) | 5 (38.5 %) |
| *Other* | 4 (10 %) | 2 (16.7 %) | 2 (13.3%) | 0 (0 %) |
| **ER-percentage (%)** |  |  |  |  |
| *Median (IQI)* | 100 (95 – 100) | 100 (94 – 100) | 100 (93 – 100) | 100 (100-100) |
| **Ki67** |  |  |  |  |
| *Pretreatment (IQI)* | 10 (5, 16.3) | 12.5 (4, 20) | 10 (5, 10) | 10 (10, 20) |
| *Posttreatment (IQI)* | 5 (1, 5) | 1 (1, 1) | 5 (1, 5) | 5 (5, 20) |
| **NET duration (months)** |  |  |  |  |
| *Median (IQI)* | 7.2 (6.6 – 8.0) | 7.5 (6.9 – 7.9) | 7.7 (6.7 – 8.4) | 6.7 (5.8 – 7.2) |
| **Type of NET** |  |  |  |  |
| *Tamoxifen* | 12 (30 %) | 2 (16.7 %) | 5 (33.3 %) | 5 (38.5 %) |
| *Aromatase inhibitor* | 23 (57.5 %) | 8 (66.7 %) | 8 (53.3 %) | 7 (53.8 %) |
| *Combination* | 5 (12.5 %) | 2 (16.7%) | 2 (13.3 %) | 1 (7.7 %) |
| **Pretreatment CPE** |  |  |  |  |
| *Median (IQI)* | 0.30 (0.25 – 0.44) | 0.27 (0.21 – 0.36) | 0.31 (0.26 – 0.43) | 0.32 (0.26 – 0.51) |
| **Pathologic response (%)** |  |  |  |  |
| *Complete response* | 1 (2.5 %) | 1 (8.3 %) | 0 (0 %) | 0 (0 %) |
| *Partial: <10% tumor* | 2 (5%) | 2 (16.7%) | 0 (0%) | 0 (0%) |
| *Partial: 10-50% tumor* | 16 (40%) | 5 (41.7%) | 6 (40%) | 5 (38.5%) |
| *Partial: >50% tumor* | 16 (40%) | 4 (33.3%) | 7 (46.7%) | 5 (38.5%) |
| *No response* | 5 (12.5 %) | 0 (0 %) | 2 (13.3 %) | 3 (23.1 %) |
| Unless otherwise specified data are number of patients, with percentages in parentheses. CPE = contralateral parenchymal enhancement, IQI = interquartile interval, IDC = invasive ductal carcinoma, ILC = invasive lobular carcinoma, ER = estrogen receptor, NET = neoadjuvant endocrine therapy. | | | | |
